# Urea, and the Methods Employed for Its Quantitative Determination*Read before the Medical Society of the County of Albany, April 3d, 1878.

**Published:** 1878-07

**Authors:** Willis G. Tucker

**Affiliations:** Professor of Inorganic Chemistry in the Albany Medical College


BUFFALO

Medical and Surgical Journal.

VOL. XVII. JULY, 1878. No. 12.

### Original Communications.

—:o:—

ART. I.—*Urea: and the Methods employed for its Quantitative Determination.**—By WILLIS G. TUCKER, M. D., Professor of Inorganic Chemistry in the Albany Medical College.

Urea is a stable chemical compound of definite composition and the most important product resulting from the destructive metamorphosis of the nitrogenous tissues of the body. It is the chief organic constituent in the urine of man. Its formula is $C\,H_4\,N_2\,O$, and it is isomeric with ammonic cyanate, by the spontaneous transformation of which it may be produced.

In most of the books it is stated that it was discovered by Rouelle in 1773, but Thudichum has shown that it was in reality first obtained by Boerhaave more than half a century before that time. Boerhaave evaporated a large quantity of urine; put it aside in a closed vessel and noticed that after a time a mass of colored crystals formed in the liquid. These he removed; dissolved in water, and, after filtration and evaporation obtained a "white purified salt," which, to quote from the translation of his *Method of Chemistry*, was "of a nature different from all other salts, . . . . so that it may justly be called *the essential salt of*

*Read before the Medical Society of the County of Albany, April 3d, 1878.

*the urine*, or of the human body." There can be no doubt, therefore, that the honor of the discovery belongs to the Dutch physician and not to the French chemist. Rouelle, however, got the credit. After the publication of his results it was obtained in a state of greater purity by Fourcroy and Vauquelin, and later Berzelius prepared it in colorless crystals, as did also Prout, who established its composition. Wohler obtained it artificially from ammonic cyanate in 1828, and for this artificial preparation Liebig subsequently gave a formula.

Pure urea may be obtained from the urine by evaporation and extraction with alcohol, or by precipitation with nitric acid and the subsequent decomposition of the nitrate formed. Artificially it may be prepared by the decomposition of potassie cyanate by ammonie sulphate, the ammonic cyanate which is formed being converted into urea on evaporation.

In a pure state urea may be kept indefinitely without undergoing change, but if brought into contact with certain animal substances it undergoes decomposition and is converted into ammonic carbonate according to the following reaction :

$$C\,H_4\,N_2\,O + 2\,(H_2\,O) = (N\,H_4)_2\,C\,O_3.$$

This decomposition occurs spontaneously in stale urine, and may also take place within the bladder.

For many years after its discovery urea was believed to be formed in the kidneys, because they were regarded as true glandular organs and its presence could not be shown in the blood. With the introduction of better methods of analysis, however, its constant presence in the blood was shown, being about .16 of one per cent. There is about twice the quantity in the blood of the renal artery that there is in that of the renal vein. Urea is found also in considerable quantity in the lymph and chyle, and in small quantity in the sweat, vitreous humor, the substance of the liver and the muscular juice.

As to the origin of urea it seems most consistent with observed facts to suppose that it is formed, not alone by the disassimilation of the nitrogenized tissues, but also by the decomposition of the surplusage of similar substances taken as foods. As to the source first mentioned, while it is undoubtedly impossible to show the presence of anything but minute quantities of urea in the tissues there is little difficulty in believing that it is washed out from them by the lymph and blood as fast as formed, and it has been experimentally shown that after the removal of the kidneys from dogs and rabbits an accumulation of urea takes place in the liver, muscles and brain. As regards the second source it is held by some authorities that there is nothing to show that the nitrogenized principles of food may ever be transformed directly into urea, but if this is not the case it is surprising that the influence of a purely animal diet shall be to increase the amount of urea eliminated, since a mixed diet is the more nourishing of the two. That some urea may also be formed in the blood by the decomposition of uric acid is possible, and Neubauer has shown that its administration to rabbits causes a direct increase in the amount of urea excreted.

The total amount of urea eliminated daily by a healthy adult ranges from 450 to 600 grains, and is liable to considerable variation. It constitutes from two to three per cent. of the urinary secretion, but the the quantity will depend upon the nature and amount of food consumed, or if little or no sustenance be taken upon the amount of nitrogenized matter disintegrated in its place. It is generally admitted to be increased by active exercise and, according to Hammond, by unusual mental exertion.

In conditions of disease the amount of urea eliminated may be largely increased or diminished. Transient increase or decrease may be due to temporary apathy of the kidneys or to accidental polyuria, but the continued excretion of an amount larger or smaller than the average becomes a sign of disease. An increase in the excretion of urea accompanies all febrile diseases, with perhaps a single exception, yellow fever. The quantity may rise to from 75 to0 1200 grains per day, or to double the normal quantity. As febrile symptoms abate the amount sinks, falling sometimes below the normal quantity, but during convalescence it rises to the natural amount. In epilepsy the excretion of urea is generally large, and in diabetes it may reach a thousand grains or more per day. In twenty-nine cases reported by Harley it averaged 728

grains. In diabetes insipidus it is also increased, and sometimes enormously. Harley gives the case of a man, aged fifty-six, in which it reached 1300 grains.

A diminished excretion of urea may depend upon decreased formation or upon retention. In paralytic idiocy its elimination is below the normal quantity, and in cholera it is both relatively and absolutely very small. In chronic affections generally, accompanied by defective nutrition, the amount is diminished. On the other hand, the production of urea may be normal and the amount excreted small, through defective action of the kidneys. In such cases unless proper elimination can be restored a fatal result must follow.

The analysis of urine for urea may be either qualitative or quantitative. As urea may be considered practically an invariable constituent of the urine, a quantitative analysis, if any, is generally required. This may be either roughly or accurately made. If a great excess of urea be present the addition of nitric acid to a little of the urine placed in a watch-glass will cause the formation of an abundant crop of crystals of the nitrate in flat rhombicor hexagonal plates, and by comparing the appearances as presented with different samples of urine, some idea of the relative amount of urea present may be formed.

For the accurate quantitative determination of urea numerous processes have been devised. Bunsen's method depends upon the resolution of urea into carbonic acid gas and ammonia, when it is heated in a sealed tube with a solution of barium chloride containing ammonia. The weight of the carbonic acid is calculated from that of the barium carbonate produced, and from this the ámount of urea is determined. Bunsen's process gives very accurate results, but it is too difficult for ordinary use. Ragsky and Heintz convert the urea into ammonic sulphate by the use of strong sulphuric acid, and weigh the ammonia as a chloro platinate. This method is also accurate, but difficult. Millon and Neubauer decompose the urea with mercurie nitrite. The former determines the weight of the carbonic acid gas by absorption with potash, and the latter calculates the loss of weight resulting from the escape of the carbonic acid and nitrogen together. These methods are not easy, and they are less exact than the two first mentioned. All of them require somewhat complicated apparatus, considerable manipulative skill on the part of the operator, and troublesome calculations to determine the result. The really practicable methods, adapted to the use of the physician, are Liebig's, Davy's, and that which will be last described, devised by Dr. Fowler, of New York. Liebig's method, properly performed, is the most exact in common use. It is a volumetric process, and is based upon the fact that urea is precipitated by mercuric nitrate as a white precipitate containing nitrate of urea and mercuric oxide. This process is described in all the larger works on urinary analysis. While Liebig's method is highly accurate and trustworthy it is open to certain objections, as for instance, that it requires apparatus not ordinarily possessed by the physician, no small degree of manipulative skill, and a solution of murcuric nitrate *very* carefully prepared and of exact strength. Moreover, certain precautions must be observed and certain modifications made, under peculiar circumstances, as when the amount of urea is very small, or very large, or when the urine contains carbonate of ammonia, albumen or certain other matters. While this process properly performed is perhaps the best ever devised, its employment is attended with no small amount of difficulty.

In 1854 Dr. E. W. Davy published in the *Dublin Hospital Gazette*, and about the same time in the *Philosophical Magazine*, his process for estimating urea, which, very largely from its simplicity, soon became exceedingly popular. It depends upon the fact that urea acted upon by the hypo-chlorites of soda or potash, is decomposed, being resolved into sodic chloride, water, carbonic acid gas and nitrogen, according to the following reaction :

$$C\,H_4\,N_2\,O+3\,Na\,Cl\,O = 3\,Na\,Cl+2\,H_2\,O+C\,O_2+N_2.$$

Dr. Davy found that the carbonic acid gas liberated does not escape in the gaseous form but remains combined with the excess of alkali in the reagent, and that the only gas which is set free is nitrogen. He determined that each cubic inch of gas liberated corresponds to .645 of a grain of urea. The directions for making the determination were given in *Thudichum's*

*Treatise on the Pathology of the Urine*, first edition, but have been omitted in the second edition of the work just issued, in which the process is slightingly referred to as "not adapted to accurate or even approximately accurate estimations." Notwithstanding this assertion it has been found largely serviceable both by professed analysts and physicians, and is still much employed. Full directions for applying the process will be found in Flint's and Pifford's manuals and elsewhere.

At a recent meeting of this Society an exceedingly convenient method for measuring the nitrogen evolved was shown by Professor Perkins, of Union University, who devised it, by which the use of mercury and the salt solution are obviated and much inconvenience avoided.

It has not been my purpose to describe the preceding methods in detail, but only to allude to them in passing, and before giving an account of a process in one sense built upon Dr. Davy's method and yet differing materially from it. In June, 1877, Dr. George B. Fowler, of New York, published in the *New York Medical Journal* an account of a "New and simple method for the quantitative estimation of urea," being the prize essay of the Alumni Association of the College of Physicians and Surgeons. The idea occurred to the author that, just as the quantity of sugar in diabetic urine may be determined by the difference in specific gravity before and after fermentation, so the same difference before and after the decomposition of urea by the hypochlorites should bear a definite relation to the quantity present in the urine. Urea consists of $\frac{7}{15}$ths of its weight of nitrogen, and therefore almost one-half will disappear. As no carbonic acid gas escapes nothing is gained from this source. It is unnecessary to describe Dr. Fowler's experiments in detail, and it need only be said that his results were found to correspond almost precisely with those obtained by Liebig's method, either when solutions of urea of known strength were employed in testing, or when similar samples of urine were operated upon. It was found that every degree of density lost corresponded to 7.791 milligrammes per cubic centimetre (about 3½ grains per fluid ounce), or to .77 per cent. Thus, if the loss in specific gravity be three degrees the urine contains 2.3 per cent. of urea, or about 10½ grains to the fluid ounce.

Experiment showed that the best hypochlorite to employ was Squibb's "Solution of chlorinated soda," or Labarraque's solution. It is superior in constancy of composition and specific gravity to any other, and is especially recommended. Seven parts by volume of this solution are capable of decomposing all the urea unless the urine contains a very great excess, in which case it should be diluted with an equal bulk of water, and the result multiplied by two.

To use this method first take the specific gravity of the urine to be examined and afterwards that of the hypochlorite. Then add to one volume of the urine seven volumes of the hypochlorite solution, when effervesence, indicating the escape of nitrogen, will ensue. Shake the jar containing the mixture occasionally, and allow it to stand for at least an hour; two hours is better. At the expiration of that time, when the liquid has become quiescent, take its specific gravity. To obtain the result we must subtract the specific gravity of the decomposed liquid from its specific gravity before decomposition; but as it is impossible to use the hydrometer upon mixing the fluids, on account of immediate effervescence, we must calculate the specific gravity of the mixed liquids. This is done by multiplying the specific gravity of the hypochlorite by seven, since we have used seven volumes, and adding to the product the specific gravity of the urine, and dividing the sum by eight. From the quotient thus obtained the specific gravity of the liquid after decomposition is to be snbtracted. The difference is the number of degrees lost, and each degree corresponds to .77 per cent. of urea.

This seems complicated, but the operation is in reality exceedingly simple. For example, if the specific gravity of the urine was 1021 and of the hyperchlorite 1045, we must add 1021 to seven times 1045 and divide the sum by eight. We obtain 1042, being the specific gravity of the mixed liquids before decomposition, and from this we subtract the specific gravity of the quieseent decomposed liquid. Suppose that is found to be 1039, then the difference is 3, which multiplied by .77 gives 2.3 as the percentage of urea in the urine tested.

The only apparatus necessary for this process is a suitable hydrometer or urinometer, a jar and a graduated vessel of convenient form for measuring the fiuid. The only chemical necessary is the solution of the hypochlorite of soda, always obtained without difficulty. For taking the specific gravities the ordinary urinometer marked up to 1060 may be made to answer, but if accuracy is required one with a longer stem and larger degrees, more easily read, is essential. Mr. John Tagliabue, of 69 Fulton street, New York, constructs a pair of hydrometers especially for this purpose. One reads from 1000 to 1030 and is used for the urine, and the other from 1030 to 1060, being used for the hypochlorite, or for the urine should the specific gravity be over 1030. Squibb's solution has an average density of 1048. The degrees on these instruments are about one-twelfth of an inch apart, and therefore the specific gravities can be very accurately taken. They cost three dollars a pair. The only objection to their use lies in the fact that a considerable quantity of liquid is required (from six to eight ounces) in which to float them, and for this reason a pair of instruments is better than a single one with a long stem. There need seldom, however, be any difficulty in obtaining the requisite quantity of urine upon which to operate, and as it is essential that the specific gravities be accurately taken a small instrument ought not to be employed.

Since changes of temperature affect the volume, and therefore the specific gravity of liquids, it is essential that the fluids shall have the same temperature when their specific gravities are taken. This is best accomplished and the use of the thermometer avoided by mixing the hypochlorite and urine in proper volume, and setting the jar aside, together with a bottle of the urine and of the hypochlorite, in the same place. When decomposition is complete the specific gravities of the three liquids can be taken, their temperatures being of course the same, and the calculation easily made. Neither sugar nor albumen interfere with this test.

This process is undoubtedly highly accurate, and it is certainly so simple as to commend itself to the favor of the busy practitioner.

